# Nonbacterial Thrombotic Endocarditis With Embolic Phenomena Diagnosed by Transesophageal Echocardiogram

**DOI:** 10.7759/cureus.45686

**Published:** 2023-09-21

**Authors:** Kiriti S Vattikonda, Christopher J Peterson, Vira I Ayzenbart, Molly S Rutherford

**Affiliations:** 1 Internal Medicine, Virginia Tech Carilion School of Medicine, Roanoke, USA; 2 Cardiology, Virginia Tech Carilion School of Medicine, Roanoke, USA

**Keywords:** mitral regurgitation, anticoagulation, transesophageal echocardiogram, anti-synthetase syndrome, non-bacterial thrombotic endocarditis

## Abstract

Nonbacterial thrombotic endocarditis (NBTE) is a valvular disorder commonly associated with malignancy and connective tissue diseases. While the disorder is often discovered during autopsy, it is sometimes diagnosed in patients who present with systemic embolization. Here, we discuss the case of a 52-year-old female, with connective tissue disease and malignancy, who presented with symptoms of systemic embolization and was diagnosed with NBTE by transesophageal echocardiogram (TEE). This case highlights the utility of TEE in diagnosing NBTE and its influence in guiding the subsequent management of patients.

## Introduction

The pathophysiology of non-bacterial thrombotic endocarditis (NBTE) was first described by Zeigler in 1888 as the presence of vegetation composed of fibrin and platelet aggregates with the absence of inflammation or bacteria [1]. Initially, NBTE was referred to as marantic, derived from the Greek word “marantikos” meaning “wasting away”, and in 1923 as terminal endocarditis by Libman [2]. Gross and Friedberg described the pathology more extensively and coined the term "nonbacterial thrombotic endocarditis" in 1936. Another group of authors Allen and Sirotas used the term “degenerative verrucous endocarditis” and thought the vegetation was from degenerated valve collagen, rather than thrombosis. This term was largely ignored in the literature until 1954, when Angrist and Marquis presented evidence that these valvular lesions resulted in systemic emboli. Shortly thereafter, MacDonald and Robbins confirmed the evidence and further highlighted the clinical relevance of emboli, not just in patients with carcinoma or debilitating disease [3]. Non-invasive imaging modalities guide clinicians in the premortem diagnosis of NBTE when embolic phenomena are present, whether as clinically apparent or occult.

Among the various pathologies affecting heart valves, NBTE encompasses a broad spectrum of noninfectious lesions. It is commonly seen in advanced malignancy and connective tissue disorders with clinical presentations of patients including life-threatening thromboembolism to the brain, heart, spleen, kidney, and other vital organs. NBTE is often found in autopsies at the rate of 0.9-1.6% [4]. A variety of factors may predispose a patient to NTBE, including circulating immune complexes, hypoxia, hypercoagulability, and carcinomatosis [1]. However, some patients can be diagnosed while living when they present with evidence of systemic embolization requiring therapy. As in infectious endocarditis, echocardiography is crucial to making the diagnosis and is often the first-line imaging modality [5], although high suspicion may be needed when an initial transthoracic echocardiogram (TTE) is negative. Indeed, additional imaging modalities such as transesophageal echocardiography (TEE) may be required. Correctly diagnosing NBTE is crucial as emboli can occur in a high percentage of cases (mean 42%) [6]. Misdiagnosis can be devastating and result in worse outcomes from persistent emboli [7]. In some cases, the addition of a TEE was crucial to making the correct diagnosis [8]. Here, we describe such a case requiring a follow-up TEE to diagnose NBTE.

## Case presentation

A 52-year-old female with a history of anti-synthetase syndrome, interstitial lung disease, recent diagnosis of antiphospholipid syndrome complicated by embolic stroke, and severe mitral regurgitation (MR) on prior TTE presented to the hospital after a short (two to three minutes) episode of atypical chest pain. The chest pain occurred at rest and lasted only a few minutes. High sensitivity troponin peaked at 495 ng/L (normal <37 ng/L). Electrocardiogram showed normal sinus rhythm and left atrial enlargement. A review of systems revealed intermittent vision loss since the stroke despite apixaban therapy. She was treated with a loading dose of aspirin (324 mg) followed by daily maintenance of 81 mg and continuous heparin infusion. 

**Figure 1:**
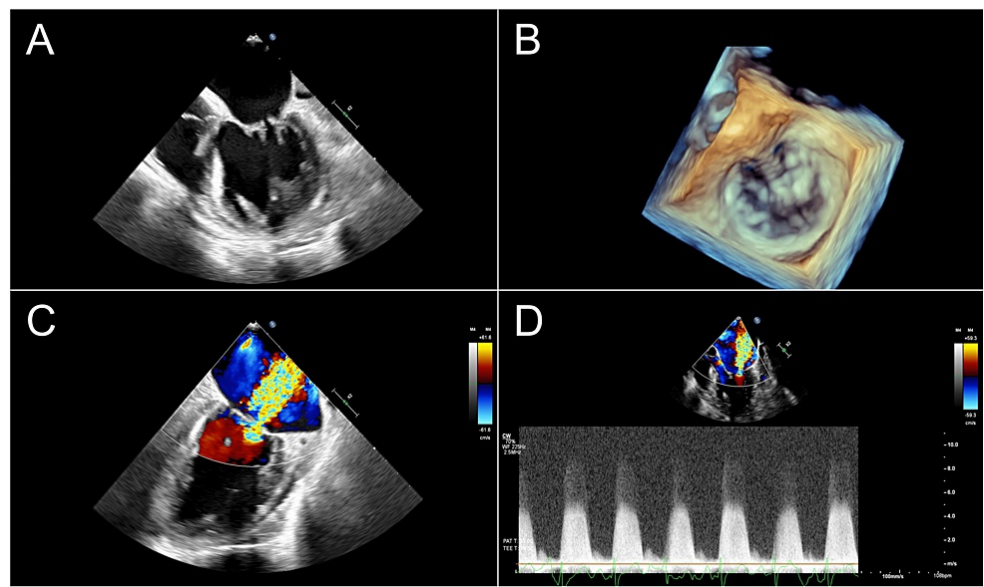
TEE imaging showing (A) mitral valve thickening with small free-moving vegetations, and (B) 3D rendering with posterior mitral valve leaflet restriction due to vegetations. (C) Doppler and (D) color Doppler studies demonstrating severe mitral regurgitation. TEE: transesophageal echocardiography

To evaluate the patient's chest pain, complicated by coronavirus disease 2019 (COVID-19) and hemoptysis, we performed CT angiography (CTA) instead of percutaneous angiography of the coronaries. The CTA showed a coronary calcium score of zero and a new 13 mm right lower lobe peripheral nodule. A subsequent positron emission tomography (PET) scan was done to evaluate the nodule and showed increased glucose uptake in that nodule along with mediastinal and hilar lymph nodes. Given her reported embolic symptoms, a TEE was pursued to better elucidate the contributory origin of her symptoms. The TEE demonstrated severe mitral regurgitation, posterior mitral valve leaflet restriction, and mitral valve thickening with small free-moving vegetation (Figure 1).

TEE proved to be the key diagnostic tool in this case as negative blood cultures, absence of fevers, normal C-reactive protein, and vegetation confirmed on TEE supported the diagnosis of nonbacterial endocarditis. She was started on warfarin (INR goal 2-3) with subsequent resolution of vision changes and other embolic symptoms attributed to NBTE. The patient was discharged with plans for follow-up with cardiothoracic surgery, pulmonology, and hematology-oncology.

The patient subsequently underwent surgery for mechanical mitral valve replacement, left atrial appendage resection with primary closure, and mediastinal lymph node biopsy two months after discharge with an adjustment of warfarin INR goal (2.5-3.5). The pathology of the excised mitral valve showed fibrosis and myxoid degeneration while the lymph node pathology noted non-small cell lung carcinoma (NSCLC). Her NSCLC was treated with a combination of chemotherapy (carboplatin and paclitaxel) and radiation. Surveillance PET scan was without any clear sign of progressive disease. As of the time of this writing, the patient has had no further episodes of thrombi or emboli. 

## Discussion

NBTE is characterized as the presence of sterile vegetations on heart valves, in contrast to the more common infectious endocarditis [9]. Damaged cardiac valves result in platelet aggregation and thrombi formation [1]. It is most frequently associated with malignancy, hypercoagulable states, immune complex diseases, hypoxia, and systemic inflammatory conditions, including malignancy, rheumatoid arthritis, and antiphospholipid syndrome [1,5]. Libman-Sacks endocarditis, a subset of NBTE, is specific to patients with systemic lupus erythematosus. Rates of NBTE are highest amongst patients with malignancy and connective tissue disorders such as SLE [4,10,11].  

Diagnosis of NBTE involves the identification of a thrombus via imaging and the exclusion of infectious endocarditis [1]. The tendency for smaller thrombi formation in NTBE (75%, <3mm) formation may evade detection on TTE [1]. Embolization may further decrease vegetation size and limit detection [5]. There are several studies comparing TTE and TEE, which unsurprisingly show greater sensitivity with TEE [4].  For example, in a 20-year single-center retrospective study, TEE identified 40.5% of cases (19/45) compared to 97.1% by TEE (33/34; p<0.01), with an identification of 21 cases by TEE but not TTE [5]. A study of 81 patients with SLE received initial TTE followed by TEE, with TEE showing greater sensitivity for valve vegetations, valve thickening, aortic regurgitation, and any valve abnormality (5%, 52%, 6%, and 57% vs. 46%, 70%, 14%, and 70%, respectively; p < 0.05) [12].

Treatment for NBTE typically involves anticoagulation, with low-molecular-weight heparin or unfractionated heparin preferred. Although warfarin is typically discouraged in patients with malignancy due to recurrence, it may be considered for long-term coagulation in patients with autoimmune and inflammatory disorders [13,14]. In this case, the patient's previous failure while on apixaban and anticipated mitral valve replacement supported warfarin use. Direct thrombin or factor Xa inhibitors have not been evaluated for NBTE. Indications for surgical management are similar to those for infective endocarditis, though it must nonetheless be individualized given the lack of formal guidelines [14,15].

This diagnosis explained the embolic shower to cerebral, ocular, and coronary arterial territories resulting in a stroke, amaurosis fugax, and non-ST elevation myocardial infarction, respectively. Etiology is likely a combination of malignancy (she was subsequently diagnosed with NSCLC) and auto-immune disorders (anti-synthetase syndrome). Furthermore, the diagnostic information provided by the TEE also significantly influenced management as we switched the patient to warfarin from apixaban, given the failure of the previous therapy. 

## Conclusions

NBTE should be investigated in patients with hypercoagulable states complicated by systemic embolism. NBTE presents a major diagnostic challenge for physicians given the wide range of contributory etiologies. In this clinical vignette describing embolic phenomena from NBTE, we provide additional evidence to suggest direct factor Xa inhibitors in addition to direct oral anticoagulants may not effectively prevent thromboembolic events. The superior image quality available with TEE was critical in diagnosing our patient with NBTE and identifying the source of emboli. This case stresses the importance of combining TEE imaging with astute clinical reasoning to make a difficult diagnosis in a complex patient, guide treatment decisions, and improve patient outcomes.
